# The need to change: Is there a critical role of midlife adaptation in mental health later in life?

**DOI:** 10.7554/eLife.82390

**Published:** 2023-05-04

**Authors:** Friederike Thams, Stefanie Brassen

**Affiliations:** 1 Department of Systems Neuroscience, University Medical Center Hamburg-Eppendorf Hamburg Germany; State University of New York Upstate Medical University United States; State University of New York Upstate Medical University United States

**Keywords:** late-life depression, successful aging, developmental model, emotion regulation, adaptation, midlife

## Abstract

Although late-life depression (LLD) is a serious health problem and more common than dementia in people over 60, it is underdiagnosed and undertreated. The cognitive-emotional etiology of LLD is particularly poorly understood. This is in contrast to the now extensive literature from psychology and cognitive neuroscience on the characteristics of emotionally healthy aging. This research consistently shows a change in emotional processing in older adults that is modulated by prefrontal regulation. Lifespan theories explain this change in terms of neurocognitive adaptation to limited opportunities and resources that typically occur in the second half of life. Epidemiological data on an increase in well-being after a low point around age 50 suggest that the majority of people seem quite capable of making this adaptation, even though empirical evidence for a causal modulation of this so called ‘paradox of aging’ and for the role of the midlife dip is still lacking. Intriguingly, LLD is associated with deficits in emotional, cognitive, and prefrontal functions similar to those shown to be crucial for healthy adaptation. Suspected causes of these deficits, such as white matter lesions or affective instability, become apparent as early as midlife when internal and external changes as well as daily challenges set in. Based on these findings, we propose that some individuals who develop depression at older ages may not have been able to successfully implement self-regulatory adaptation at midlife. Here, we review the current evidence and theories on successful aging, the neurobiology of LLD, and well-being across the lifespan. Drawing on recent advances in lifespan theories, emotion regulation research, and cognitive neuroscience, we propose a model of successful versus unsuccessful adaptation that emphasizes the increasing need for implicit habitual control and resource-based regulatory choice during midlife.

## Introduction

When it comes to health problems in older age, modern medicine focuses primarily on Alzheimer’s dementia (a Medline search over the last 10 years using the terms ‘Alzheimer’s disease/dementia’ yielded about 105k publications), while late-life depression (LLD) receives much less attention (using the terms ‘late-life depression/geriatric depression’ about 11k publications were found). One of the main reasons for this could be that many patients, but also general practitioners, still consider depression as a normal side effect of aging (Park and Unützer, 2011). In clinical practice, typical symptoms of depression in older people, such as insomnia, loss of appetite, social isolation, and reduced activity levels, are often (mis)attributed to aging (Allan et al., 2014). In addition, older adults’ attitudes toward treatment, including perceived stigma or ageism (viewing depression as a normal part of aging), may discourage them from actively seeking help for mental illness (Nair et al., 2020). As a result, although LLD is more common than dementia in people over 60 years of age, with prevalence rates of 8–29% (Naismith et al., 2012; Horackova et al., 2019), it is underdiagnosed and undertreated (Allan et al., 2014). This can have dramatic consequences, as LLD typically follows a chronic-remitting course, is related to an increased risk of suicide and decreased physical, cognitive, and social functioning (Blazer, 2003). All of these are associated with increased mortality, more frequent use of healthcare, and significantly higher healthcare costs (Bock et al., 2017).

**Figure 1.**
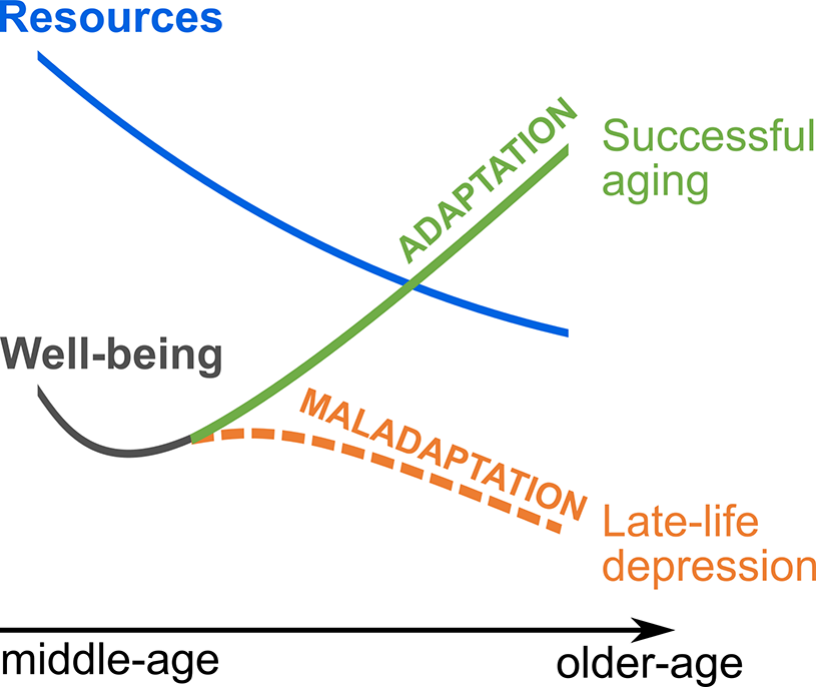
Well-being in older age as a proposed function of midlife adaptation to resources.

Most emphasis has been placed on the importance of biological changes in LLD (Alexopoulos et al., 1997; Naismith et al., 2012), while there are few theories on psychocognitive etiology (but see Blazer, 2003; Fiske et al., 2009). Important insights into the cognitive-emotional development of LLD may be provided by research on successful aging, which has linked emotionally healthy aging to successful neurocognitive and psychological adaptation to age-related limitations (Carstensen, 2006; Park and Reuter-Lorenz, 2009; Brassen et al., 2012; Mather, 2016; Heckhausen et al., 2019). Such limitations in physical, cognitive, and socioeconomic functioning as well as time perspective become increasingly evident from midlife onward (Carstensen, 2006; English and Carstensen, 2014; Heckhausen et al., 2019), which points to midlife as a pivotal phase in the life course of well-being (Lachman et al., 2015). Accordingly, first longitudinal studies support the predictive value of midlife functioning for mood state and depression later in life (Dennerstein et al., 2004; Lachman et al., 2015; Campbell et al., 2020). This suggests that some individuals who develop depression at older ages may not have been able to successfully use self-regulatory adaptation strategies at earlier ages (as schematically shown in Figure 1), raising important questions about target modulators and critical time windows for prevention and early intervention (Richmond-Rakerd et al., 2021). Below, we review current evidence on the neurobiological and psychocognitive features of LLD and successful aging, as well as epidemiological data on well-being across the lifespan. Incorporating these findings with assumptions on emotion regulation from cognitive neuroscience, we propose a neurocognitive developmental model of emotionally successful and unsuccessful (i.e. LLD) aging that focuses on the critical role of cognitive-emotional adaptation and prefrontal function in midlife.

### Neurocognitive features of late-life depression

There is considerable heterogeneity in LLD with respect to neuroanatomical, cognitive, clinical, and genetic profiles, which recently has led to the delineation of at least two-dimensional representations (Wen et al., 2022). In a narrow sense, LLD is referred to as late-onset depression (LoD), which differs from early-onset depression (EoD) by an illness onset not before the age of 60 (but sometimes 55 or 65). This distinction is reflected in unique clinical features and pathophysiological pathways to illness. For example, LLD has been associated with the absence of a family history of affective disorders, cognitive deficits, more somatic symptoms, and less personality dysfunction (Fiske et al., 2009; Naismith et al., 2012). Although we focus primarily on LoD in this review, it is important to note the limitations of this categorical distinction. In the clinical context, it can be very difficult to retrospectively determine the exact time of onset, and earlier depressive symptoms may have escaped formal medical diagnosis and treatment (Naismith et al., 2012). Furthermore, EoD may also promote later vascular depression through its negative effects on vascular health, inflammation, and epigenetics (Alexopoulos, 2019). In any case, the identification of specific LLD subgroups has helped to delineate specific pathophysiological profiles of LLD, some of which are likely to arise in midlife (Alexopoulos, 2019; Moura et al., 2019) and are associated with cognitive deficits (Sierra et al., 2004) that may pave the way for clinical symptoms later in life.

#### Structural disruption of fronto-subcortical networks

The most striking neurobiological feature of LLD is the disruption of fronto-subcortical structure. Reported networks include the dorsolateral prefrontal cortex (dlPFC), ventromedial PFC (vmPFC), anterior cingulate cortex (ACC), basal ganglia (caudatus, putamen), amygdala, and hippocampus (for comprehensive reviews, see Naismith et al., 2012; Kim and Han, 2021). A recently published ALE (activation likelihood estimation) meta-analysis of 17 structural imaging studies with LLD patients reveals the most consistent pattern of decline to be located in the ACC and medial prefrontal cortex compared to age-matched healthy controls. The authors also applied a novel coordinate-based network mapping approach that additionally showed the involvement of frontoparietal control, dorsal attention, and visual networks, particularly in late-onset LLD (Zhukovsky et al., 2021).

Several, partially overlapping etiological factors for these disruptions have been discussed, including vascular mechanisms, inflammation, neuroimmune regulatory dysfunction, neurodegenerative changes, and amyloid accumulation (for recent reviews, see Alexopoulos, 2019; Jellinger, 2022). Another highly consistent finding in LLD is the observation of white matter lesions (WMLs), which are recognizable as hyperintense on T2-weighted images and which are predominantly caused by ischemic changes in small vessels (van Agtmaal et al., 2017). The observation of WMLs together with common vascular risk factors and cerebrovascular disease (Geraets et al., 2022) have led to the ‘vascular depression’ hypothesis (Alexopoulos et al., 1997), which posits that cerebrovascular disease predisposes and/or maintains depression in later life. WMLs are mainly localized in subcortical structures and their frontal projections such as the cingulum bundle (Taylor et al., 2003). Their burden is directly related to symptom severity (Kim and Han, 2021) and reduced cognitive control ability (Köhler et al., 2010) in patients with LLD as well as in (yet) nondepressed older (Mayda et al., 2011) and middle-aged (Sierra et al., 2004) individuals. Moreover, WML burden in LLD is associated with an elevated BOLD response in the subgenual ACC (sgACC) during an affective-reactivity task (Aizenstein et al., 2011). WMLs predict incident depression over time (van Sloten et al., 2015), and can occur as early as the mid-40s (Moura et al., 2019), and thus can be considered as a strong risk and vulnerability factor for LLD.

#### Cognitive-emotional alterations

Consistent with structural alterations of networks mediating cognitive control, (Tadayonnejad et al., 2014; Zhukovsky et al., 2021), patients with LLD often show severe deficits in executive functions such as cognitive flexibility, planning, response-inhibition or set-shifting (Herrmann et al., 2007; Naismith et al., 2012), which led to the conceptualization of the ‘depression executive dysfunction syndrome’ (Alexopoulos, 2019). Cognitive deficits, that also include impairments in processing speed as well as in learning and memory, are associated with a problematic prognostic and clinical perspective (Alexopoulos et al., 2002).

Although affected prefrontal regions and related higher-order cognitive functions are typically involved in emotion regulation, that is, control of limbic activity (Ochsner et al., 2012; Etkin et al., 2015, see below for further details), surprisingly few studies have focused specifically on emotional processing or regulation in LLD. Existing behavioral findings suggest a cognitive bias toward negativity (Broomfield et al., 2007; Brassen et al., 2008; Aizenstein et al., 2011; Huang et al., 2019; Baruch et al., 2021), similar to the well-described negativity or mood-congruency effect in younger patients (Mennen et al., 2019; Robinson, 2019). In accordance with this, task-based functional magnetic resonance imaging studies show neural changes in response to emotional inputs in LLD compared to nondepressed older adults in the dlPFC, vmPFC, rostral, and sgACC, and the amygdala-hippocampus complex (Brassen et al., 2008; Brassen et al., 2012; Aizenstein et al., 2011; Briceño et al., 2015; Wong et al., 2016; Leal et al., 2017; Vasudev et al., 2018; Huang et al., 2019), suggesting dysfunctions in emotion regulation, particularly in response to negative stimuli. For example, compared to emotionally healthy older adults, patients with LLD show heightened behavioral and autonomic regret responsiveness when being confronted with missed opportunities in a sequential risk-taking task (Brassen et al., 2012). Effects are paralleled by a reduced engagement of the vmPFC which may reflect the failing of automatic or implicit downregulation of negative stimulation in LLD (Suri and Gross, 2012). Intriguingly, emotionally healthy older adults show reduced regret responsiveness not only compared to LLD patients but also to healthy young adults, and this effect is associated with increased vmPFC engagement (Brassen et al., 2012). Findings indicate an age-related shift in emotion regulation that may help healthy older adults to overcome regretful thoughts, that, if left unresolved, can eventually lead to intense brooding and depressive symptoms (Aldao et al., 2010).

### Neurocognitive features of emotionally healthy aging

LLD is the most common mental disorder in later life (Volkert et al., 2013). On the other hand, three out of four older adults describe themselves as having aged successfully (Bowling and Dieppe, 2005). Epidemiological data even show that older people report better emotional well-being compared with younger adults (Stone et al., 2010; Blanchflower, 2021), and this holds true under the COVID-19 pandemic (Carstensen et al., 2020). What are the mechanisms for this so-called ‘paradox of aging’ (Mather, 2012; Carstensen, 2019), given that older people face frequent losses, increasing physical and psychosocial limitations, and the perception that lifetime is running out?

#### Lifespan theories

To answer this question, lifespan psychologists, neuroscientists, and epidemiologists have identified resilience factors that may promote successful aging (Carstensen, 2006; Park and Reuter-Lorenz, 2009; Brassen et al., 2012; Mather, 2016; Heckhausen et al., 2019). The concept of successful aging is hereby often equated with emotional health (Badache et al., 2023), although it has undergone different interpretations since its introduction in the early 1960s (Havighurst, 1961). There are three well-known psychobiological models of successful aging. The first is the selection, optimization, and compensation (SOC) model (Baltes and Baltes, 1990), which assumes that successfully aging individuals use these three concepts to set, pursue, and maintain realistic personal goals in the face of age-related declines in resources. The second model, the motivational theory of lifespan development (Heckhausen et al., 2010; Heckhausen et al., 2019), extends this model by emphasizing individuals’ lifelong motivation to maximize personal agency over their environment (‘primary control’). It is hypothesized that this primary control is achieved through resource-oriented adaptation of goal selection, goal engagement, and goal disengagement. Secondary control strategies involve self-regulation techniques to either ensure motivational commitment during goal engagement or to facilitate goal disengagement and self-protection when goals or primary control cannot be achieved. Third, the socioemotional selectivity theory (Carstensen, 2006), which focuses specifically on the role of remaining lifetime perception for adaptive shifts in emotional goals and strategies across the lifespan. That is, when people perceive their time as expiring, they shift in focus from future to present and increasingly invest cognitive control in regulating emotional states to optimize their well-being in the short term. All three theories thus highlight the need for aging individuals to adapt to limited resources in goal setting and goal pursuit.

In their SOC-ER framework, Urry and Gross, 2010, embed these assumptions in the emotional context by combining SOC (Baltes and Baltes, 1990) with the process model of emotion regulation (Gross, 1998). Specifically, they argue that older people select and optimize particular emotion regulation strategies as a consequence of available internal (e.g. cognitive) and external (e.g. environmental) resources (Urry and Gross, 2010). Evidence for these assumptions is provided by findings on emotion processing observed in healthy older adults.

#### Cognitive-emotional changes

The most consistent finding related to emotionally healthy aging can be subsumed as a positivity effect (PE), reflected in either increased processing of positive or decreased processing of negative information in older compared to younger adults. Such prioritization of positive over negative information has been documented in the domains of attention, memory, and decision-making (Reed et al., 2014; Mather, 2016). Due to the typical use of extreme age-group designs, a meta-analysis on more than 100 studies on the PE report mean age distances between groups ranging from 45 to 55 years (Reed et al., 2014). The size of the PE thereby positively scales with the magnitude of the age distances within studies, which argues for gradual changes across the lifespan.

The PE has been associated with increased emotional stability (Brassen et al., 2011) and better immune function (Kalokerinos et al., 2014). Accordingly, emotionally healthy older people appear to engage differently in emotion regulation compared to healthy young (Gross et al., 1997; Nolen-Hoeksema and Aldao, 2011; but see Isaacowitz, 2022) and older depressed individuals (Brassen et al., 2012). Older people may even become better at regulating their emotions (Urry and Gross, 2010). For example, they may be more effective in situation selection (Gross, 1998), as indicated by longitudinal data showing the quantitative (but not qualitative) narrowing of social networks from midlife on to improve emotional experience in everyday life (English and Carstensen, 2014). Emotionally healthy older adults deploy more attention to positive than to negative information in order to improve (Isaacowitz et al., 2008) and stabilize (Brassen et al., 2011) their mood. They are also more effective in focusing away from negative emotions (Phillips et al., 2008), and downregulating negative emotions is perceived as less costly (Scheibe and Blanchard-Fields, 2009). In contrast, older adults are less successful at cognitively demanding reappraisal, particularly detached reappraisal (Shiota and Levenson, 2009). This shift away from explicit, cognitively demanding, toward less costly, attentional, and mainly implicit (see below) emotion regulation probably results from reduced cognitive capacity (Opitz et al., 2012) and may reflect successful adaptation in terms of SOC (Urry and Gross, 2010). It should be noted that age-effects on emotion regulation have not been consistently observed, with some studies reporting no age differences or even suggesting age-similarity in emotion regulation (e.g. Eldesouky and English, 2018; Livingstone and Isaacowitz, 2019). However, previous evidence points toward differences in effectiveness and frequency of use of certain strategies in young and older adults (e.g. Scheibe and Blanchard-Fields, 2009; Shiota and Levenson, 2009), which may be related to age-associated neural changes. In a similar vein, a recent review suggests that older adults’ brains may automatically engage in behavior leading to positive emotions through action-value signaling from vmPFC/ventral ACC promoting such behavior (Isaacowitz, 2022). Several neuroimaging findings support this idea, which are presented in the next section.

#### Prefrontal modulation of emotion regulation in aging

Neuroimaging findings on emotion regulation in successful aging indicate a key role of the vmPFC/ACC. For example, the strength of vmPFC-amygdala coupling at rest predicts the occurrence of a positivity bias in memory (Sakaki et al., 2013), there is an increased ACC/vmPFC activity during selectively focusing on positive information (Leclerc and Kensinger, 2008; Brassen et al., 2011), and activity in the ACC underlying an attentional PE is directly related to emotional well-being in older age (Brassen et al., 2011). In addition, increased activation in the vmPFC in response to negative information is associated with enhanced striatal (Brassen et al., 2012) and reduced amygdala signaling (Corbett et al., 2020), underlining the critical role of the medial PFC in modulating emotion processing in healthy aging.

The key role of the vmPFC in emotion regulation is based on its unique position to integrate information about current context, goals, motivational states, and learning history to compute and update the subjective value of stimuli and orchestrate appropriate responses to them (Roy et al., 2012). Information about the relevance of stimuli to affective goals is provided mainly by subcortical regions such as the amygdala and the ventral striatum (Haber and Knutson, 2010), whereas current goals can be represented by more lateral prefrontal regions (Lapate et al., 2022). It has been speculated that the vmPFC functionally compensates for the decline of the dlPFC in healthy aging (Mather, 2016). Indeed, whereas lateral prefrontal brain regions typically undergo strong age-related decline, the function and structure of the vmPFC remains relatively well preserved across the lifespan (Fjell et al., 2009). Direct support for this idea is provided by findings that increased activity of the vmPFC in response to negative stimuli correlates with lower lateral PFC function and structure in healthy older adults (van Reekum et al., 2018).

It has further been speculated that pronounced vmPFC engagement reflects a shift toward implicit emotion regulation in healthy aging (Suri and Gross, 2012). Indeed, most studies demonstrating a PE have not provided explicit instructions for regulating emotions and most findings could be attributed to an attentional bias (Mather and Carstensen, 2003; Isaacowitz et al., 2008; Brassen et al., 2011; Brassen et al., 2012). However, the effects of cognitive effort and habitual control on the PE clearly need further investigation (Dolan and Dayan, 2013; Petro et al., 2021). Studies involving cognitive manipulation tend to support the relevance of cognitive resources in promoting top-down regulation (Kryla-Lighthall and Mather, 2009; Brassen et al., 2011; Brassen et al., 2012; Mather, 2012; Sasse et al., 2014). For example, an attentional PE in older adults during an emotional interference task occurred only when more attentional resources were available (Brassen et al., 2011), and focusing on positive and ignoring negative information was directly related to executive control function in a nonemotional visual search task in older participants (Sasse et al., 2014). On the other hand, studies on early processes and habituation suggest that bottom-up processes are also involved in the emergence of emotional selectivity in older persons (Gronchi et al., 2018; Petro et al., 2021). Most likely, both implicit and explicit processes orchestrate age-related adaptation of emotional behavior as a function of neurocognitive resources, compensatory abilities, and lifelong learning. The underlying framework of implicit and flexible regulation is briefly introduced in the next section.

### Cognitive neuroscience of emotion regulation

The selection and pursuit of goals is thought to rely on habitual and flexible control (Cushman and Morris, 2015). Applied to an emotional context, this could mean that an individual habitually seeks to avoid negative situations and approach positive ones (habitual control), but needs to employ a range of flexible (cognitively controlled), complex strategies (e.g. distract from an emotional stimulus or engage in reappraisal) to achieve this goal, depending on the specific situation and one’s resources (Raio et al., 2016).

Nevertheless, implicit processes can be involved in all stages of emotion regulation, from goal selection over monitoring to regulation selection and enactment (Koole et al., 2015). For instance, the goal to prioritize emotional well-being and meaning may first be consciously adopted but later be implicitly activated as a form of stimulus-response pairing by an emotionally relevant context or cue (Williams et al., 2009). Whether or not to engage in emotion regulation is then determined by monitoring divergences of emotional responses from this goal, that, at least in part, may also occur on implicit levels and has been related to the vmPFC/ventral ACC (Etkin et al., 2006; Etkin et al., 2015; Egner et al., 2008). Once emotion regulation is warranted, strategy selection can also rely on implicit processes. Specifically, habitual strategies, formed by frequent and consistent use and linked to certain situations (if-then implementations), can be elicited automatically when dealing with undesired emotions (Christou-Champi et al., 2015). For instance, an aging person may learn to lower her expectations each time she needs to compete physically or cognitively with younger persons, to prepare for possible failure. Such implicit emotion regulation may also bias attention toward stimuli that may help to achieve goals (Vogt et al., 2011), which is particularly interesting given the frequent finding of an attentional positivity bias observed in emotionally health older adults (Brassen et al., 2011; Reed et al., 2014; Sasse et al., 2014). On the neural level, implicit regulation has been consistently associated with activation in the ventral/rostral ACC and the vmPFC (Quirk et al., 2006; Egner et al., 2008; Brassen et al., 2011; Etkin et al., 2015).

Implicit regulation may be cognitively less demanding but at the cost of behavioral flexibility. In novel situations where it becomes necessary to adjust behavior to changing internal and external requirements, flexible planning may be more appropriate (Cushman and Morris, 2015). Cognitive flexibility is a major component of cognitive control, and implicates different PFC regions, including the lateral PFC and orbitofrontal cortex (Friedman and Robbins, 2022). The choice of explicit, goal-directed emotion regulation depends heavily on the individual’s ability to weigh the costs and benefits of applying or refraining from control. In other words, engaging in cognitively costly strategies (e.g. reappraisal, Buhle et al., 2014) occurs when the expected benefits of doing so outweigh the costs (Kool et al., 2018). The capacity for both cost-benefit analysis and cognitively demanding explicit strategies mainly relies on prefrontal function, with the dorso- and ventrolateral PFC playing a particular role (Smittenaar et al., 2013; Buhle et al., 2014; Cortese, 2022). Given pronounced age-related changes in the (dorso)lateral PFC (Fjell et al., 2009; Cabeza and Dennis, 2013), it seems likely that how and when people engage in such cost-benefit trade-offs change across the lifespan. Indeed, older adults show an increase in subjectively perceived cognitive costs, which reduces their motivation to engage in cognitively demanding activities (Hess et al., 2016). Interestingly, recent lifespan data suggest that when motivation is high and task requirements are manageable, older individuals are still able to selectively invest cognitive resources in cognitively costly decision-making (Devine et al., 2021; Ruel et al., 2021). These recent empirical findings fit with lifespan theories emphasizing the need of an motivation- and resource-based adaptation of goal selection, goal engagement, and goal disengagement for successful aging (Baltes and Baltes, 1990; Heckhausen et al., 2019).

Overall, neurobehavioral findings in successful aging suggest that there may be a shift in the balance from flexible to implicit habitual control in the second half of life. Such shift my occur as an adaptation to the high demand for emotion regulation in aging peoples’ everyday life (see below), where explicit regulation is clearly not something one can effectively engage all the time, especially in the face of declining resources. But when does such adaptation occur? Adaptation in self-control is clearly a lifelong process but probably particularly critical in midlife, not only because it is a phase of fundamental internal and external changes but also because individuals are still capable to build aging preparedness (Lachman et al., 2015; Richmond-Rakerd et al., 2021).

### Midlife challenges and risk factors for maladaptation

Large cross-sectional epidemiological surveys can provide a detailed look at emotional trajectories and potentially critical time windows for adaptation across the lifespan. They consistently show a U-shaped pattern of subjective well-being with an average nadir around age 50. This midlife nadir corresponds to the peak of unhappiness in the late 40s across Europe and the United States (Blanchflower, 2020; Blanchflower, 2021). The dip in well-being fits the hypothesized window of midlife crisis, originally described as the period when we face our limitations, restricted possibilities, and mortality (Jaques, 1965). The U-shape has been observed in more than 140 developing and developed countries and holds regardless of whether sociodemographic covariates or cohort effects are included (Blanchflower and Oswald, 2008; Stone et al., 2010; Blanchflower, 2021; Kaiser et al., 2022).

Although midlife is considered a critical period for preparing for the demands of aging (Richmond-Rakerd et al., 2021), compared with earlier and later life stages, the factors that mediate well-being in the middle years are less well understood (Lachman et al., 2015), nor is it known whether a potential low point is predictive of later mood. Mediators of well-being at midlife that have been discussed include the approaching of physical, cognitive, and socioeconomic demands as well as age discrimination by employers, financial pressures, limited opportunities, and changing family dynamics such as parental caregiving and empty-nest transitions (although children leaving home has also been discussed as a later positive factor for well-being because of lower levels of family conflict and financial burden; Stone et al., 2010). For a comprehensive overview and discussion of midlife conceptions, see Lachman et al., 2015.

The ability to deal with these challenges may be compromised by neuronal and related cognitive changes that are increasingly setting in from midlife on (Park and Festini, 2016). Specifically, white matter integrity of prefrontal-subcortical connections starts to degenerate significantly between 40 and 50 years of age (Gunning-Dixon et al., 2009; Yeatman et al., 2014). White matter decline may be further accelerated by WMLs, which first peak in the mid-40s (Moura et al., 2019) and which are then already associated with cognitive decline (d’Arbeloff et al., 2019). Pronounced occurrence of WMLs may be driven by an increase of cardiovascular dysfunctions or inflammatory processes associated with metabolic diseases such as obesity, which also become more prevalent during midlife (Power et al., 2017; Mattson and Arumugam, 2018). Similarly, gray matter changes have been related to cognitive decline, particularly in executive functions (Ferreira et al., 2014; Park and Festini, 2016), which can be observed as early as age 40 (Singh-Manoux et al., 2012; Ferguson et al., 2021). Cognitive abilities are most important for successful adaptation (Cañas et al., 2003). Deficits in cognitive control are related to the use of maladaptive emotion-regulation strategies (Joormann and Gotlib, 2010) and increase individuals’ vulnerability for the first onset of depression (Gotlib and Joormann, 2010). Cognitive capacity is relevant not only for the successful use of cognitively demanding strategies (e.g. reappraisal), but also for the flexible selection of adequate strategies in light of changing resources (Urry and Gross, 2010; Heckhausen et al., 2019).

Thus, long-established regulation strategies (e.g. reappraisal; active undoing of regrettable decisions) may no longer be sufficiently usable due to decreased resources (Heckhausen et al., 2019). Adequate stress regulation may be further compromised by hormonal changes (Hodes and Epperson, 2019), with potential neuronal consequences due to the downstream effects of strong corticosteroid exposure (Kiesow et al., 2021). The increasing awareness of the aging mind and body, limited opportunities, and decreasing time horizon (Carstensen, 2006) may be particularly crucial for well-being as people in middle age tend to review their ‘first half’ of life (Ingersoll-Dayton et al., 2010). If they then perceive low controllability (Lachman et al., 2015; Robinson and Lachman, 2017) to effectively cope with missed opportunities (e.g. raise a child, get a career promotion), this can severely impact self-efficacy, agency, and well-being now and later (Blazer, 2002; Wrosch and Heckhausen, 2002). The crucial role of midlife self-control for well-being has recently been demonstrated in a longitudinal study that measured the impact of self-control in a population-representative cohort that was followed from birth to age 45. Not only childhood self-control but also self-control in midlife was hereby associated with pace of aging and the preparedness to manage later-life health, financial, and social demands, even after accounting for self-control, IQ, or socioeconomic origins in childhood. These data indicate that self-control in midlife is a malleable target for intervention but may also be a target for disruption (Richmond-Rakerd et al., 2021).

There are only few longitudinal studies of midlife predictors for depression in later life. Most of them focus on women and reveal that negative mood, negative attitudes toward aging, affective instability, chronic stress, as well as less optimism in nondepressed midlife adults predict later depression (Dennerstein et al., 2004; Lachman et al., 2015; Campbell et al., 2020). For example, lower positive mood scores and higher stress levels at 50 years is associated with higher reporting of depressive symptoms for women when they were aged 70 years (Campbell et al., 2020), suggesting that higher positive mood and effective stress regulation during the transition from midlife into late life may be a resilience factor for emotional health in aging. That is, empirical data may indicate negative effects of a midlife mood low on later well-being. This fits with assumptions that dysthymic disorder can persist from midlife into late life (Blazer, 2003), or findings on the predictive value of midlife mood disturbance for later inflammation, which in turn is a strong risk factor for LLD (Alexopoulos, 2019).

### A neurocognitive model of emotional adaptation in the second half of life

**Figure 2.**
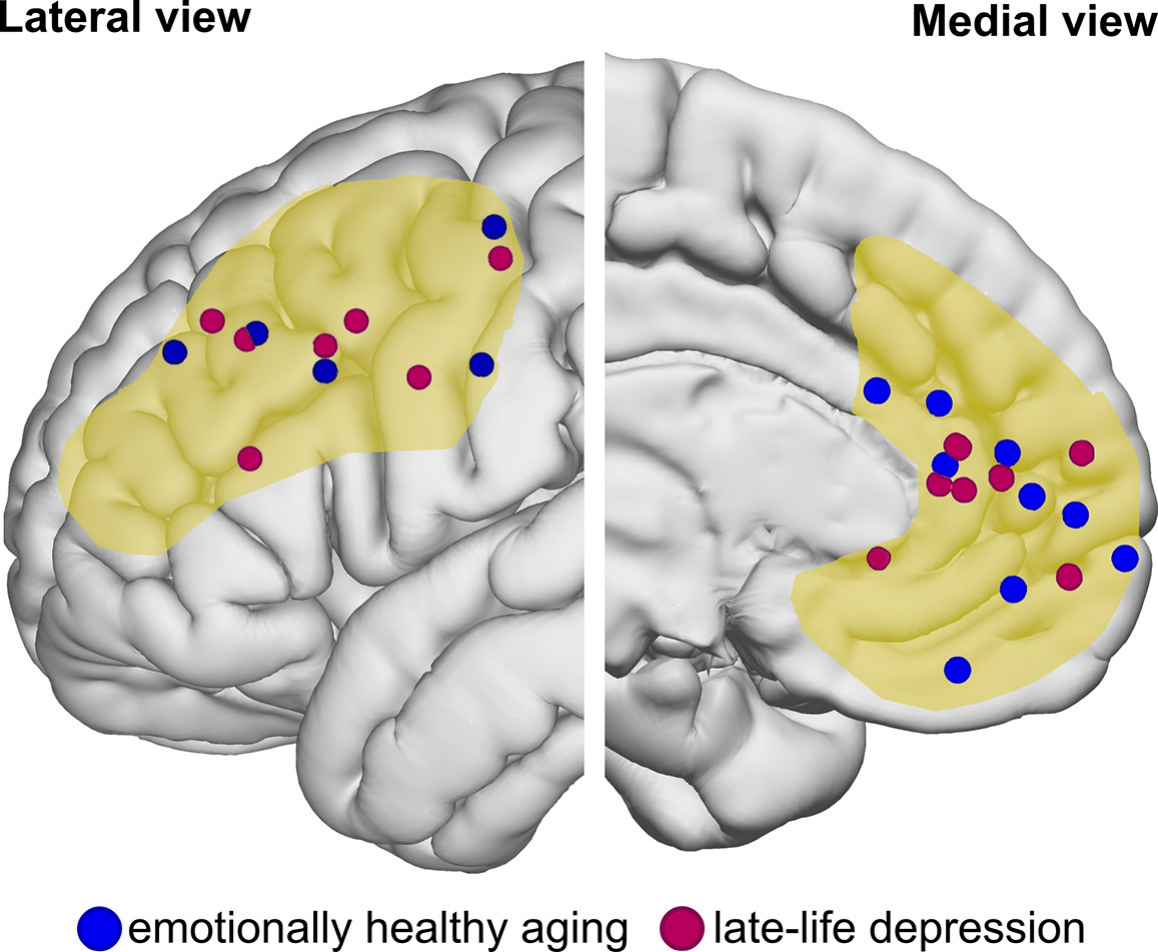
Prefrontal activation foci from studies in emotionally healthy and late-life depressed older adults. Blue dots represent foci related to prefrontal activation differences during emotional processing found in emotionally healthy older compared to healthy younger adults. Magenta dots represent foci related to prefrontal activation differences during emotional processing found in late-life depressed compared to nondepressed older adults. Based on the focus of this review, foci falling within the boundaries of the dorsolateral and ventromedial prefrontal cortex (illustrated in yellow) are shown if peak coordinates were provided by the individual studies. Selected studies are summarized in Supplementary file 1.

The previous sections show that there is a striking overlap in the neurobehavioral factors involved in emotionally healthy aging and in LLD. First, there is the PE in healthy aging (Reed et al., 2014), which contrasts with a negativity bias in LLD (Broomfield et al., 2007). Second, the PE is reflected in prefrontal engagement, particularly in the vmPFC/ventral ACC (Brassen et al., 2011; Mather, 2016), which in turn is functionally altered in response to negative information in patients with LLD (Brassen et al., 2008; Brassen et al., 2012; Aizenstein et al., 2011). Third, the structure of the vmPFC is relatively well preserved in healthy aging (Fjell et al., 2009; Mather, 2016), whereas it is one of the key regions that exhibits structural and functional disruption in LLD (Naismith et al., 2012; Zhukovsky et al., 2021). Fourth, higher-order cognitive functions appear to be involved, at least in part, in the development of a PE (Fischer et al., 2010; Mather and Knight, 2005; Murty et al., 2009; Ritchey et al., 2011; Roalf et al., 2011; Sasse et al., 2014) and are needed for flexibly dealing with new challenges (Friedman and Robbins, 2022). Executive functions and dlPFC are strongly affected in LLD compared to older controls (Naismith et al., 2012; Alexopoulos, 2019). An overview of the prefrontal regions reported to be critical for emotionally successful aging and LLD is provided in Figure 2.

Based on this overlap, we hypothesize that some patients with LLD have not adapted successfully in emotional goals and strategies as suggested by lifespan theories. We propose that individuals who missed a resource-adapted change in emotion regulation during midlife are at increased risk to develop depression in later life. Midlife is a period when many people first confront approaching endings, limited opportunities, and aging brains and minds. To maintain self-efficacy and well-being in the face of restricted time perspective, motivational goals are shifted toward prioritizing short-term, emotionally meaningful goals over exploration and long-term growth. The explicitness of this goal shift is unclear, but it is likely that it is, at least in part, driven by an implicit adaptation to gradual internal and external changes. This goal shift leads to an increasing focus on pro-hedonic emotion regulation in daily life which may be guided by an implicit attentional bias toward stimuli supportive for goal achievement.

Ideally, strategy selection can rely on both habitual and flexible control, depending on the novelty and demands of the specific situation. For the implementation of habitual control, people have to learn over the years which strategy is most effective in a given context and then automatize strategy use so that it becomes more effective and less cognitively costly (Scheibe et al., 2021). In situations in which habitual control is not possible or sufficient (e.g. sudden life-event such as loss of a parent, retirement), individuals should select regulation strategies based on available resources, including biological and societal opportunities and constraints as well as preexisting regulatory skills. Such cost-benefit analysis is mediated by the degree of goal motivation that determines individuals’ willingness to invest cognitive and time resources in regulatory strategies (Devine et al., 2021; Ruel et al., 2021). Successful application of a selected regulation strategy may – after sufficient repetition – pave the way for automation of that strategy (e.g. habitual reappraisal).

Given the potentially high demand for daily regulation in midlife to cope with mostly chronic challenges, we postulate a need for a shift to less costly, well-implemented, implicit regulation to maintain emotional stability and self-efficacy in the second half of life. This is particularly necessary given the decline in cognitive flexibility and underlying neural processes that typically occur in normal aging. When the specific situation requires flexible action, limited resources should be used only on the basis of a rational cost-benefit analysis. Thus, we conclude that successful emotional adaptation in the second half of life depends on (i) lifelong acquired skills/regulatory implementations and underlying networks mediating implicit habitual control, that is, vmPFC/ventral ACC, and (ii) sufficient resources for the flexible use of regulatory strategies when required, which may then transform into implicit processes in the longer term as resources become more limited. This flexible control in turn depends on appropriate functioning of dorsolateral PFC regions.

**Figure 3.**
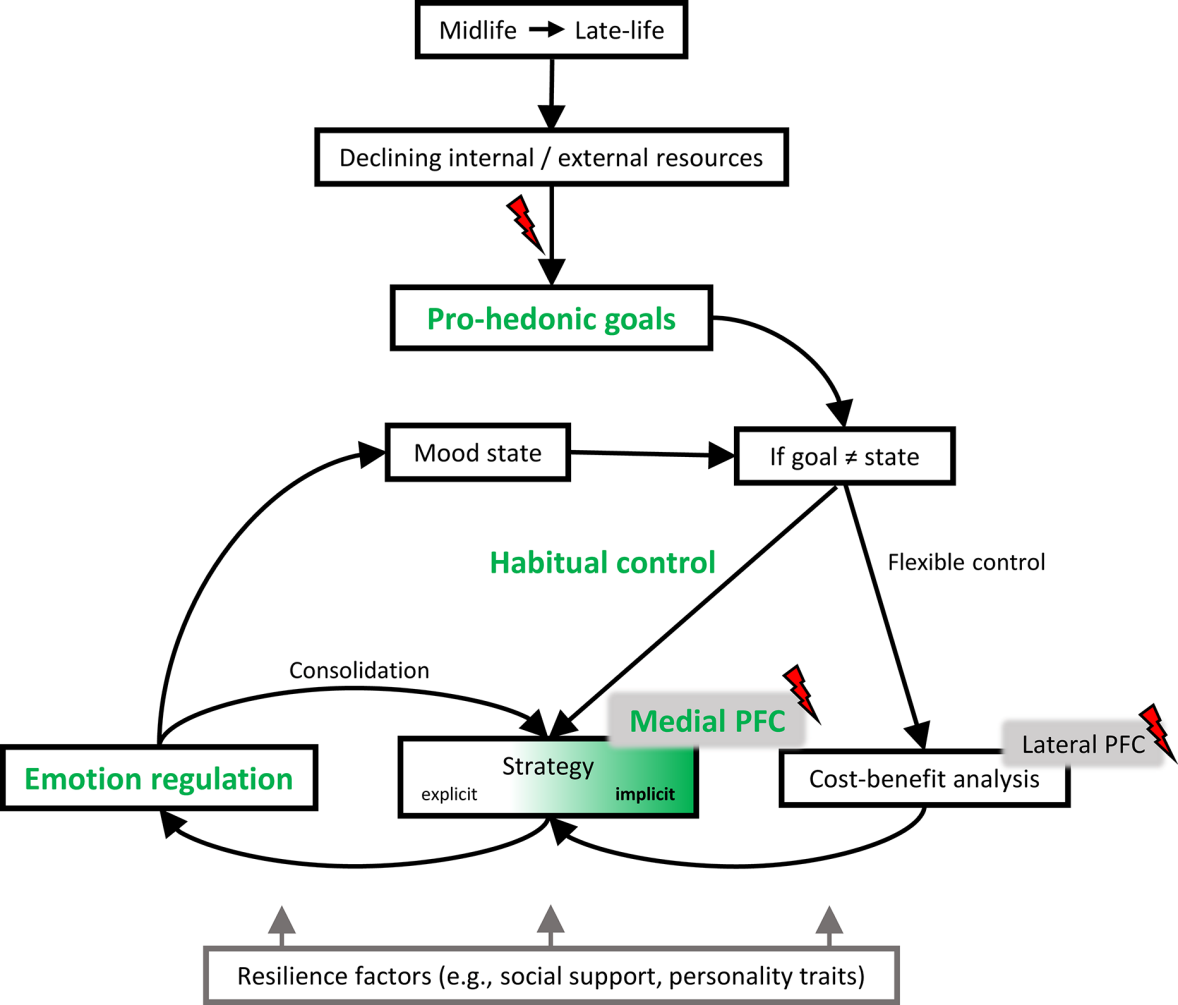
A neurocognitive model of emotional adaptation in the second half of life. Proposed framework on successful transition of emotion regulation from mid- to late-life. Green font indicates aspects of specific importance for successful emotional aging. Red lightning bolts mark aspects vulnerable to midlife risk factors for late-life depression (LLD) development.

Accordingly, we propose that one or both of these implicit and explicit regulatory processes are impaired in midlife individuals at risk for LLD. Potential reasons include (i) skill deficits, stress hypersensitivity, and maladaptive self-regulation as reflected in a preexisting cognitive (negativity) bias, for example due to childhood adversity or personality traits (pessimism, neuroticism); (ii) deficits in implicit learning and regulation due to pronounced disruption of medial PFC networks, for example due to vascular or inflammatory factors; (iii) pronounced deficits in cognitive flexibility and the underlying lateral PFC networks. Our model assumptions are summarized in Figure 3.

Several behavioral and neurobiological predictions can be derived from this model. For example, drawing on reinforcement learning approaches, one might expect gradual transitions from model-based to model-free or exploratory to exploitative decision-making from midlife to late-life, to be related to the functional and structural integrity of ventromedial prefrontal networks which may compensate for dlPFC decline, and may be associated with emotional health and well-being. Of particular interest may be paradigms that directly link decision-making to validated, non-monetary, emotional outcomes such as regret (Brassen et al., 2012). There is debate about whether and how age affects emotion regulation (Isaacowitz, 2022). Our model highlights a shift from flexible/explicit to habitual/implicit processes in emotional goal adaptation, regulation activation, and strategy in healthy aging that has rarely been considered in this debate due to the nature of typical study designs. Such a shift should now be tested directly using explicit and implicit task conditions, the latter of which should include evaluation of the degree of implicitness, for example through post hoc assessments of individuals’ awareness of strategy use. Ultimately, longitudinal studies combining well-controlled experimental tasks and neuroimaging with naturalistic, epidemiological investigations of individual differences in habitual strategy use and perceived costs could provide important insights into the proposed shifts from midlife to older age.

### Conclusions

We proposed a conceptual framework for understanding the need for emotional adaptation in midlife to maintain emotional health and self-efficacy later in life by integrating research on successful aging and LLD with assumptions from cognitive neuroscience. We postulate that the habitual activation of computationally less costly implicit emotion regulation becomes more and more important in midlife, when many individuals face cognitive, emotional, and time constraints while at the same time the demands of day-by-day challenges increase. Risk factors for pronounced disruption of the neural networks mediating implicit emotion regulation have been described in patients with LLD as early as midlife, which, together with potentially preexisting maladaptation, may hinder some individuals from shifting toward pro-hedonic goals and habitual control. In addition, there is the need to adapt flexible control strategies to changing internal and external resources by relying on a resource-based selection of emotion regulation. Accordingly, pronounced deficits in cognitive control and underlying lateral prefrontal brain regions, which are typically observed in LLD and start to set in during midlife, may represent another risk factor for missing adaptation. Within this conceptual framework, it may now be possible to construct task conditions under which the interaction between habitual and flexible emotion regulation can be studied in populations from critical age and mood states. So far, most research on successful aging used extreme age-group designs, ignoring the crucial and heterogeneous middle-aged group. In addition, small samples often do not allow for systematic analysis of multidimensional resilience or risk profiles, for example, in terms of brain function and compensatory mechanisms, vascular or regulatory strategies. Recent efforts to conduct epidemiological studies with large sample sizes covering a wide age range and follow-up assessments will certainly help to overcome these issues and may provide new insights into critical time windows and target modulators for the early intervention and prevention of the most common mental disorder in later life. The appropriate selection of emotion regulation, taking into account available resources, may hereby be a particularly promising target for modulation in midlife, when sufficient resources are still available to implement less costly strategies that can then compensate for advanced deficits and prevent at-risk individuals from becoming depressed later in life.

## Additional files

Supplementary file 1. Selected studies from Figure 2.
