# Supplementary material for: The need to change: Is there a critical role of midlife adaptation in mental health later in life?
Source: eLife. 2023 May 4;12:e82390. doi: 10.7554/eLife.82390 (PMC10159621; doi:10.7554/eLife.82390)
Supplement: Supplementary file 1. [file elife-82390-supp1.docx]

**Supplementary file 1**

**Studies in emotionally healthy and late-life depressed older adults with prefrontal activation foci (illustrated in Figure 2).**

| **Study** | **Task** | **Peak** |  | **MNI coordinates** | | |
| --- | --- | --- | --- | --- | --- | --- |
|  |  |  |  | **x** | **y** | **z** |
| **Late-life depressed patients vs. healthy older controls** | | | | | | |
| Brassen et al., 2008 | emotional spatial cueing task | medial OFC | L | -6 | 57 | -6 |
|  |  | sup ACC | L | -9 | 36 | 9 |
|  |  | pre ACC | R | 12 | 39 | 15 |
| Brassen et al., 2012 | sequential risk taking task | pre ACC | L | -20 | 44 | -4 |
| Briceño et al., 2015 | evaluation of emotional faces | SFG | R | 20 | 38 | 38 |
|  |  | MFG | L | -21 | 20 | 34 |
|  |  | MFG | R | 24 | 15 | 38 |
| Huang et al., 2019 | emotional Stroop task | SFG | L | -20 | -8 | 48 |
|  |  | pre ACC | R | 4 | 40 | 8 |
| Mah et al., 2011 | evaluation of emotional faces | MFG | L | -40 | 5 | 29 |
|  |  | MFG | R | 41 | 32 | 16 |
| Vasudev et al., 2018 | evaluation of emotional words | medial SFG | L | -3 | 59 | 14 |
|  |  | pre ACC | L | -12 | 46 | 10 |
| Wang et al., 2008 | emotional oddball task | SFG | R | 25 | 32 | 35 |
| **Emotionally healthy older adults vs. emotionally healthy younger adults** | | | | | | |
| Brassen et al., 2012 | sequential risk taking task | pre ACC | L | -20 | 44 | -4 |
| Brassen et al., 2011 | evaluation of emotional words | sup ACC | L | -12 | 36 | 22 |
|  |  | MFG | L | -42 | 32 | 36 |
| Corbett et al., 2020 | valence encoding task | SFG | R | 18 | 66 | -3 |
|  |  | medial OFC | R | 3 | 39 | -21 |
| Fischer et al., 2010 | learning of emotional faces | MFG | R | 27 | 44 | 32 |
| Leclerc &  Kensinger, 2010 | emotional visual search task | medial SFG | R | 7 | 51 | 7 |
| Murty et al., 2009 | learning of emotional scenes | MFG | L | -38 | -7 | 53 |
|  |  | MFG | L | -35 | -5 | 31 |
| Opitz et al., 2012 | gaze-directed reappraisal task | sup ACC | L | -6 | 26 | 24 |
| Ritchey et al., 2011 | evaluation of emotional pictures | sup ACC  pre ACC | L  L | 19  -8 | 37  47 | 12  14 |
| Roalf et al., 2011 | evaluation of emotional pictures | MFG | L | -35 | 20 | 30 |
| Sakaki et al., 2013 | learning of emotional faces | medial SFG | L | -6 | 58 | 4 |
| van Reekum et al., 2018 | evaluation of emotional scenes | medial OFC | R | 6 | 48 | -8 |

Regions defined according to the automated anatomical labelling atlas AAL3 (Rolls et al., 2020). Reported Talairach coordinates were transformed to MNI coordinates using the BioImage Suite Web Tool (<https://bioimagesuiteweb.github.io/webapp/mni2tal.html>).
ACC: anterior cingulate cortex, pre: pregenual, sup: supracallosal, MFG: middle frontal gyrus. OFC: orbitofrontal cortex, SFG: superior frontal gyrus. L: left, R: right.
